# Combined Simulation of a Micro Permanent Magnetic Linear Contactless Displacement Sensor

**DOI:** 10.3390/s100908424

**Published:** 2010-09-09

**Authors:** Jing Gao, Wolfgang F.O. Müller, Felix Greiner, Dirk Eicher, Thomas Weiland, Helmut F. Schlaak

**Affiliations:** 1 Graduate School Computational Engineering, TU Darmstadt, Dolivostraße 15, D-64293 Darmstadt Germany; 2 Computational Electromagnetics Laboratory (TEMF), TU Darmstadt, Schloßgartenstraße 8, D-64289 Darmstadt Germany; E-Mails: mueller@temf.tu-darmstadt.de (W.M.); thomas.weiland@temf.tu-darmstadt.de (T.W.); 3 Institute of Electromechanical Design (EMK), TU Darmstadt, Merckstraße 25, D-64283 Darmstadt Germany; E-Mails: f.greiner@emk.tu-darmstadt.de (F.G.); d.eicher@emk.tu-darmstadt.de (D.E.); schlaak@emk.tu-darmstadt.de (H.S.)

**Keywords:** PLCD sensor, contactless displacement measurement, electromagnetic simulation, thermal analysis

## Abstract

The permanent magnetic linear contactless displacement (PLCD) sensor is a new type of displacement sensor operating on the magnetic inductive principle. It has many excellent properties and has already been used for many applications. In this article a Micro-PLCD sensor which can be used for microelectromechanical system (MEMS) measurements is designed and simulated with the CST EM STUDIO^®^ software, including building a virtual model, magnetostatic calculations, low frequency calculations, steady current calculations and thermal calculations. The influence of some important parameters such as air gap dimension, working frequency, coil current and eddy currents *etc.* is studied in depth.

## 1. Introduction

**Figure 1.**
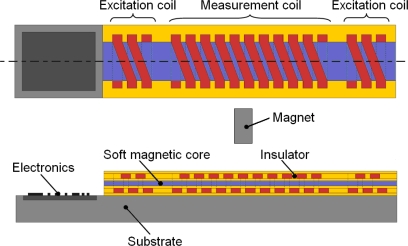
Design model of the Micro-PLCD sensor.

The measurement of displacement or position is one of the most important and oldest tasks in sensor technology [1]. As a novel magnetic displacement sensor, the PLCD sensor is characterized by continuous, contactless, linear displacement measurement, long lifetime, high reliability *etc.*, and has already been used for many applications in the automotive, hydraulic press, container security and food industries [2]. In order to realize its application in MEMS large scale absolute displacement measurement [3–5], a Micro-PLCD sensor is designed as shown in Figure 1. It consists of a soft magnetic core evenly surrounded by a coil called measurement coil. On each end of the core there is a second short coil called excitation coil which is identical and oppositely connected with each other. A permanent magnet is guided on and along the magnetic core to determine the position or displacement. Its operating principle works as follows: through two excitation coils, an excitation current causes two alternating magnetic fluxes Φ_1_ and Φ_2_ of the same size but inversely orientated in the magnetic core. If a permanent magnet of adequate strength approaches the surface of the sensor, the corresponding section of the magnetic core will reach magnetic saturation, which results in a virtual separation of the magnetic core. Then magnetic fluxes Φ_1_ and Φ_2_ will be decoupled with each other. If the permanent magnet is located above the center of the magnetic core, Φ_1_ and Φ_2_ will cancel each other with regard to the measurement coil and output voltage is zero. Any non-central position of the permanent magnet will result in a non-zero output voltage which is linearly proportional to the position of the permanent magnet.

## 2. Modeling of the Micro-PLCD Sensor

**Figure 2.**
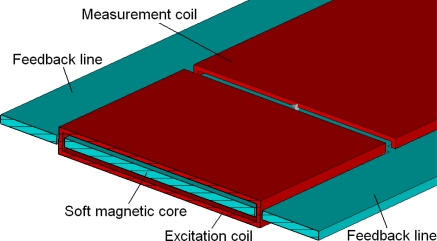
Feedback lines model.

Based on the working principle of the PLCD sensor illustrated above, this Micro-PLCD sensor is simulated and studied using Finite Integration Technique (FIT) implemented by the CST EM STUDIO^®^ software [6–8]. To improve linearity, two feedback lines are added to the soft magnetic core as Figure 2 shows. With the help of two feedback lines, the magnetic circuit of the whole sensor can form two closed loops, which can strengthen magnetic field in the soft magnetic core, increase linearity and improve output voltage of the measurement coil.

**Figure 3.**
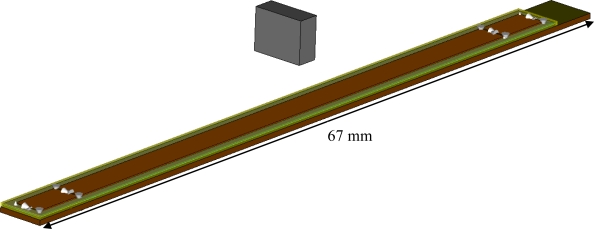
Micro-PLCD sensor model.

The whole Micro-PLCD sensor model with the permanent magnet is shown in Figure 3. The entire length of the model is designed to be between 20 mm and 77.2 mm, and the model in this paper uses 67 mm. Width and thickness of the coil is between 10 μm and 100 μm, thickness of the soft magnetic core is designed to be between 1 μm and 200 μm, and depth of the inner insulator in most cross sections is between 10 μm and 50 μm. Most of the geometric data of the sensor are defined as variable parameters of CST EM STUDIO^®^ for the convenience of changing model and parameter sweeping during low frequency calculation. In this model, all functional elements are completely sealed in insulator, which makes this Micro-PLCD sensor robust to harsh environments.

**Figure 4.**
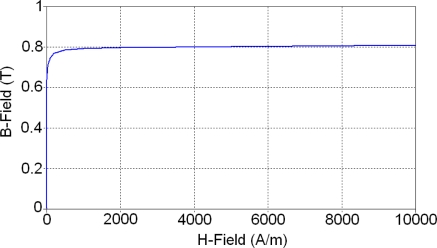
B-H curve of MUMETALL^®^.

There are mainly two different kinds of materials in the sensor. The soft magnetic core is made of a non-linear soft magnetic material. All other components such as coils, insulator, substrate and permanent magnet use linear materials and their material properties are listed in Table 1. Except for the soft magnetic material MUMETALL^®^ [9], all other materials’ relative permeability equals one. As a non-linear material, the B-H curve of MUMETALL^®^ is shown in Figure 4. It also shows that magnetic saturation induction of MUMETALL^®^ is 0.8 T; this is a very important parameter, mainly used to determine magnetic saturation regions.

Finally, all calculations in this article use electrical boundary condition, which means all simulation results below are based on the situation that the Micro-PLCD sensor works in a normal space around which it is all metal, just like it is embedded in a machine.

## 3. Introduction to the Simulation Procedure

Two different kinds of electromagnetic sources exist in this model: the permanent magnet is a magnetostatic source and two excitation coils supplied by alternating current are low frequency sources. To couple these two different sources and following thermal calculation, four calculation steps are performed successively. The magnetostatic calculation is performed first to determine the position and dimension of the magnetic saturation regions in the core and feedback line. Based on the magnetostatic calculation results, three magnetic saturation region models are built and low frequency calculations are then performed to determine the relationship between the position of the permanent magnet and the output voltage. While their relationship is proved to be linear, a simplified partial sensor model is built to calculate the current distribution, which is also a thermal source of the following thermal calculation. Finally, the temperature field distribution while the sensor is working is determined.

## 4. Magnetostatic Calculation

### 4.1. Magnetostatic calculation results

**Figure 5.**
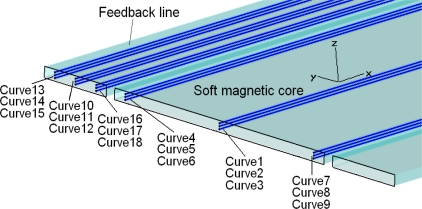
Tracking curves in the soft magnetic core and feedback line.

In order to calculate the position and dimension of all magnetic saturation regions, nine tracking curves are defined in the soft magnetic core and another nine tracking curves are defined in one feedback line, as Figure 5 shows. Magnetic flux density along these tracking curves will be calculated to determine magnetic field distribution situation in the soft magnetic core and feedback line. Because this Micro-PLCD sensor model is symmetrical along *x-z* plane (see Figure 5 for coordinate system information), there is not any tracking curves defined in the other feedback line.

**Figure 6.**
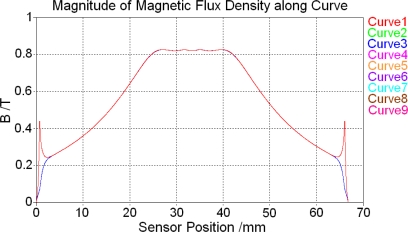
Magnetic field distribution in the soft magnetic core.

**Figure 7.**
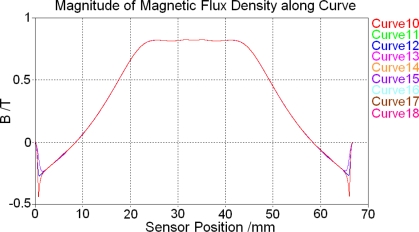
Magnetic field distribution in the feedback line.

After magnetostatic calculation is finished, magnetic flux density excited by the permanent magnet above the centre of the sensor along the nine tracking curves defined above in the soft magnetic core (curve 1 to curve 9) is shown in Figure 6. In addition, the magnetic flux density along the nine tracking curves defined above in the feedback line (curve 10 to curve 18) is shown in Figure 7.

Figure 6 and Figure 7 show that both in the soft magnetic core and feedback line, magnetic field along these tracking curves superpose over most of the sensor range. Because the magnetic circuit of the whole sensor changes direction at the two ends of the core, these result curves do not superpose at two ends. However, these ranges are very small comparing with the whole length of the sensor and located at two ends of the core, their influence can be ignored and then the magnetic field is homogeneous along *y* and *z*-direction both in the core and feedback lines. As a result there will be only one tracking curve (curve 2) left in the core and the other one (curve 11) left in the feedback line to test the magnetic field distribution situation afterwards.

**Figure 8.**
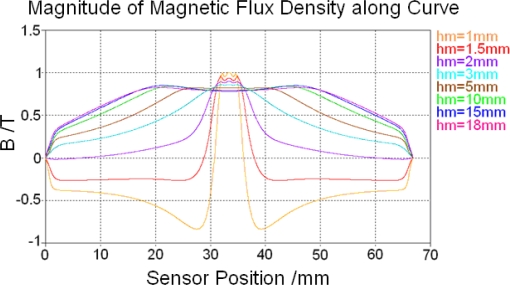
Magnetic flux density along tracking curve 2 with different *h_m_* values.

**Figure 9.**
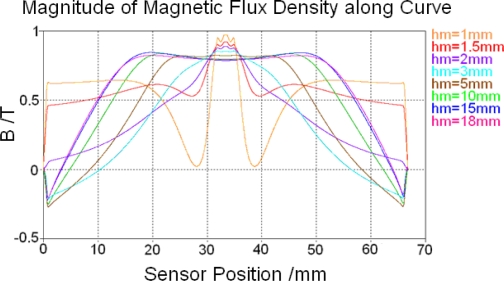
Magnetic flux density along tracking curve 11 with different *h_m_* values.

The air gap between the permanent magnet and sensor surface *h_m_* influences the magnetic saturation situation of the core and feedback lines significantly. To show its influence eight alternative values between 1 mm∼18 mm are used in simulation for reference. After magnetostatic calculation is finished, magnetic flux densities along tracking curve 2 in the soft magnetic core with different *h_m_* values are shown in Figure 8. In addition, magnetic flux densities along tracking curve 11 in the feedback line with different *h_m_* values are shown in Figure 9.

Flat tops of all resulting curves in Figure 8 and Figure 9 represent magnetic saturation regions. Their position and dimension can be measured in the software and then used for building magnetic saturation region models before low frequency calculation.

### 4.2. Magnetostatic field analysis

Figure 8 and Figure 9 also show that magnetic field of the sensor always changes direction in the soft magnetic core or feedback line. While the air gap between the permanent magnet and sensor surface *h_m_* is equal or less than 2 mm the magnetic field changes direction in the soft magnetic core and magnetic flux density’s value changes from positive to negative as shown in Figure 8, but it always remains positive in feedback lines, as shown in Figure 9. When *h_m_* is larger than 2 mm, the magnetic field changes direction in feedback lines as shown in Figure 9, but it remains always positive in the soft magnetic core as shown in Figure 8.

**Figure 10.**
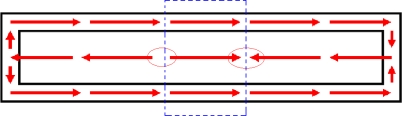
Magnetic field distribution of the whole sensor while *h_m_* = 1.5 mm.

**Figure 11.**
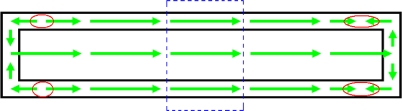
Magnetic field distribution of the whole sensor while *h_m_* = 10 mm.

To see this phenomenon more clearly, we choose two typical *h_m_* values here: 1.5 mm and 10 mm corresponding to the red result curve and green result curve in Figure 8 and Figure 9. When *h_m_* = 1.5 mm, the magnetic field distribution situation of the whole sensor is shown in Figure 10. In addition, when *h_m_* = 10 mm, it is shown in Figure 11. In these two figures, small black rectangles stand for the soft magnetic core and the area between big black rectangle and small black rectangle is the feedback line. Because the permanent magnet is located above the center of the sensor in these magnetostatic calculations, the dashed blue rectangle part of the sensor in the Figure 10 and Figure 11 is influenced by the magnetic field of the permanent magnet significantly. As a result, magnetic fields of the soft magnetic core and two feedback lines in these two blue rectangles always have the same direction with the magnetic field of the permanent magnet. Here it is always towards right. However, the soft magnetic core and two feedback lines of the sensor form two closed loops, so magnetic circuit of the sensor will also try to form two closed loops. Then magnetic field of the sensor must change its direction out of the blue rectangle to form a closed loop. While permanent magnet is very close to the sensor such as *h_m_* = 1.5 mm, magnetic field in the feedback line is larger than that in the soft magnetic core, magnetic field changes its direction near the two red circles of the soft magnetic core in Figure 10. While the permanent magnet is a little far away from the sensor such as *h_m_* = 10 mm, magnetic field in the feedback line turns smaller and then magnetic field changes its direction near the four red circles of the feedback lines in Figure 11.

## 5. Low Frequency Calculation

### 5.1. Low frequency calculation results

**Figure 12.**
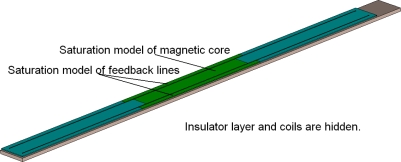
Magnetic saturation region models.

Based on the results of the magnetostatic calculation, three cuboid-like magnetic saturation region models are built in the soft magnetic core and two feedback lines with their relative permeability almost equal to unity. At the same time, the permanent magnet is ignored and the non-linear material of the soft magnetic core is simplified to a linear material. All those modifications are aimed to replace the effect of magnetic saturation phenomenon that is caused by the permanent magnet and the soft magnetic material. The position of these magnetic saturation regions will be swept from one end of the sensor to the other end with the parameter sweep function of CST EM STUDIO^®^ instead of the movement of the permanent magnet. The magnetic saturation region models located in the middle of the sensor are shown in Figure 12.

**Figure 13.**
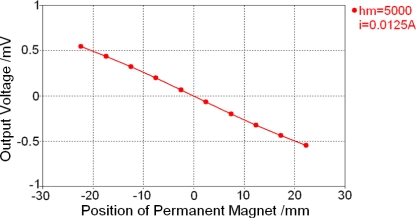
Low frequency calculation result.

After the modified model is ready, the monitor of CST EM STUDIO^®^ is set to record the voltage of the measurement coil. Finally, a low frequency calculation could be performed under the excitation of alternating excitation coil current. The relationship between the output voltage of the measurement coil and the position of magnetic saturation regions which corresponds to the position of the permanent magnet is shown in Figure 13. It shows that the relationship between the output voltage and the position of permanent magnet is linear, which proves this Micro-PLCD sensor model is a selectable design and performs very well.

### 5.2. Influence of some parameters

**Figure 14.**
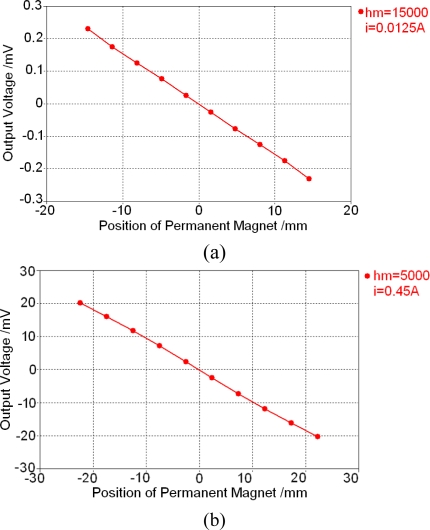
Low frequency calculation results under different working conditions. **(a)** *h_m_* = 15 mm and *i* = 0.0125A. **(b)** *h_m_* = 5 mm and *i* = 0.45A.

Figure 13 also shows that working condition of the low frequency calculation performed in the last section is: *h_m_* = 5 mm and coil current *i* = 0.0125 A, which is the minimal design value, while the maximal design value is *i* = 0.45 A. To show the influence of some important parameters, more calculations are performed and two typical results are shown in Figure 14. Their working conditions are shown in the right side of the figure.

All calculation results finished until now show that output voltage will decrease with an increment of the air gap between the permanent magnet and sensor surface but with a decrement of excitation coil current. At the same time, the relationship between the output voltage and permanent magnet can remain linear.

**Figure 15.**
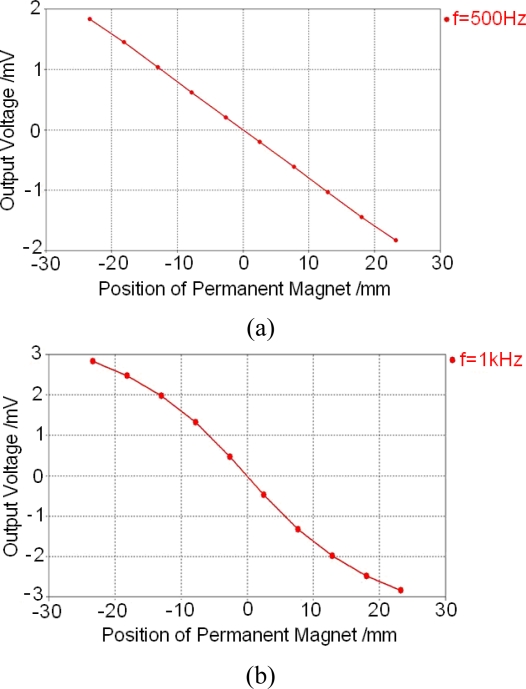
Low frequency calculation results under different working frequencies. **(a)** *f* = 500Hz. **(b)** *f* = 1 kHz.

Until now, all calculations performed before are based on a working frequency of *f* = 100 Hz. To master the influence of the working frequency, more calculations are also performed. Two typical results are shown in Figure 15.

Figure 15 and more calculations show that output voltage of the sensor will increase with the increment of the working frequency and the relationship between the output voltage and the position of the permanent magnet can remain linear when the working frequency is less than 500 Hz. Therefore, it is suggested that the working frequency of the sensor should be set to be between 100 Hz and 500 Hz.

## 6. Thermal Calculation

### 6.1. Steady current calculation results

**Figure 16.**
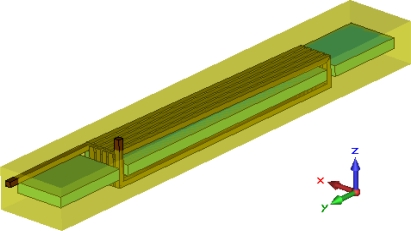
Simplified Micro-PLCD sensor model.

To obtain more information about the temperature field distribution of the whole sensor while the Micro-PLCD sensor is working, a thermal calculation is finally performed for reference. While the sensor is working, there are mainly two different kinds of currents in the sensor: coil currents and eddy currents. They are the main thermal source and make the temperature of the sensor increase. The eddy current field can be calculated based on the low frequency calculation results. In order to calculate the coil current field, a very detailed Micro-PLCD sensor model including all coil details must be used. Because the measurement coil has about 800 windings and the excitation coils have about 140 windings, a very large scale calculation model is needed for building a detail Micro-PLCD sensor model. In order to solve this problem, a simplified sensor is modelled as Figure 16 shows. In this simplified partial Micro-PLCD sensor model, a 5-turn coil sensor is modelled including the soft magnetic core, two feedback lines and the insulator. Coil current will feed in the model along the upper coil port and flow out of the model along the inferior coil port.

**Figure 17.**
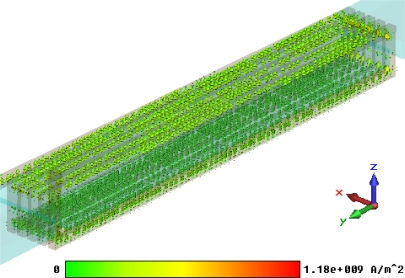
Steady current field of the partial sensor model.

Based on this simplified model, steady current calculation can be performed with the steady current solver of CST EM STUDIO^®^. The steady current field of this partial sensor model is shown in Figure 17. During this calculation the coil current is set to be a maximal value i = 0.45 A.

### 6.2. Thermal calculation results

**Figure 18.**
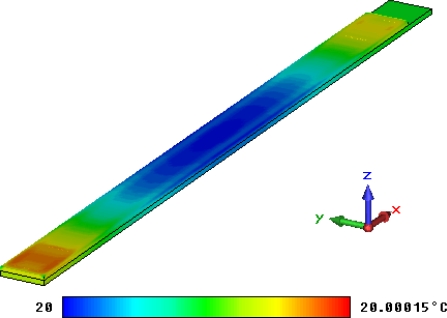
Temperature field based on eddy current.

**Figure 19.**
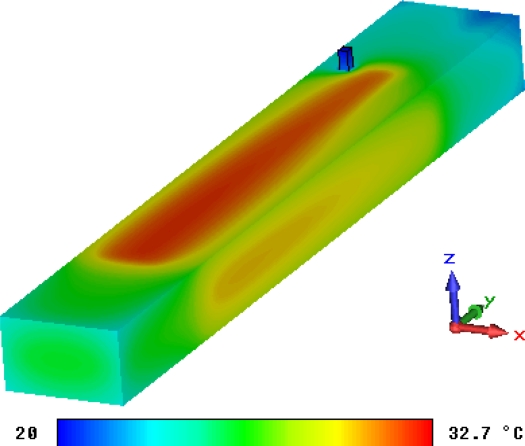
Temperature field based on coil current.

The eddy current field and the steady current field can be set as the thermal source and thermal calculations are then performed with the thermal solver of CST EM STUDIO^®^. When the coil current is set to be the maximal value *i* = 0.45 A, the temperature field distribution situation based on the eddy current is shown in Figure 18 and the temperature field based on the coil current is shown in Figure 19.

Because the eddy current field of the sensor is very small, the highest temperature in Figure 18 is almost the same as the lowest temperature and then thermal effect of the eddy current can be neglected. Figure 19 shows that the highest temperature while Micro-PLCD sensor is working is about 32.7 °C (background temperature is set to be 20 °C). Because the current of simplified model is the same as current of the excitation coil, this temperature field should be similar to the temperature field of part of the Micro-PLCD sensor corresponding to the position of the excitation coil. Apart from that, because excitation coil current should be larger than inductive current value of the measurement coil, the highest temperature in Figure 19 should be the highest temperature of the whole sensor.

## 7. Conclusions and Outlook

In this article, a Micro-PLCD sensor is designed and simulated in the CST EM STUDIO^®^ software, including magnetostatic calculation, low frequency calculation, steady current calculation and thermal calculation. All simulation results show the good performance of the designed Micro-PLCD sensor. In the near future, more parameters will be adjusted and optimized; more materials will be calculated to improve linearity and output voltage of the measurement coil. The influence of temperature, configuration of permanent magnet and sensor component, movement velocity of permanent magnet *etc.* will be studied concentratively. Real sensor samples will also be produced and tested.

## Figures and Tables

**Table 1.** Material properties of the sensor.

**Element**	**Material**	**Permittivity**	**Conductivity S/m**	**Thermal conductivity W/m·K**	**Heat capacity J/g·K**	**Density Kg/m^3^**
Coils	Copper	1	5.8 · 10^7^	401	0.385	8,960
Substrate	Silicon	11.9	1.56 · 10^−3^	148	0.703	2,329
Insulator	SU-8	3	1 · 10^−10^	0.25	1.3	1,200
Magnet	NdFeB	1	7.14 · 10^5^	18	0.45	8,700
Core	MUMETALL^®^	1	1.82 · 10^6^	18	0.45	8,700
